# Chronic binge drinking-induced susceptibility to colonic inflammation is microbiome-dependent

**DOI:** 10.1080/19490976.2024.2392874

**Published:** 2024-08-20

**Authors:** Diogo Fonseca-Pereira, Sena Bae, Monia Michaud, Jonathan N. Glickman, Wendy S. Garrett

**Affiliations:** a Department of Immunology and Infectious Diseases, Harvard T.H. Chan School of Public Health, Boston, MA, USA; b Harvard T.H. Chan Microbiome in Public Health Center, Boston, MA, USA; c Gastrointestinal Pathology, Massachusetts General Hospital, Boston, MA, USA; d Department of Pathology, Harvard Medical School, Boston, MA, USA; e Department of Molecular Metabolism, Harvard T.H. Chan School of Public Health, Boston, MA, USA; f Broad Institute of MIT and Harvard, Cambridge, MA, USA; g Division of Medical Sciences, Harvard Medical School, Boston, MA, USA; h Department of Medical Oncology, Dana-Farber Cancer Institute, Boston, MA, USA

**Keywords:** *Allobaculum*, inflammatory bowel diseases, gut microbiota, binge drinking

## Abstract

Alterations in intestinal permeability and the gut microbiome caused by alcohol abuse are associated with alcoholic liver disease and with worsening of inflammatory bowel diseases (IBD) symptoms. To resolve the direct effects of chronic ethanol consumption on the colon and its microbiome in the absence of acute or chronic alcohol-induced liver disease, we developed a mouse model of chronic binge drinking that uncovers how alcohol may enhance susceptibility to colitis via the microbiota. Employing daily ethanol gavage, we recapitulate key features of binge ethanol consumption. We found that binge ethanol drinking worsens intestinal infection, colonic injury and inflammation, and this effect persists beyond the drinking period. Using gnotobiotics, we showed that alcohol-driven susceptibility to colitis is microbiota-dependent and transferable to ethanol-naïve mice by microbiome transplantation. *Allobaculum spp*. expanded in binge drinking mice, and administration of *Allobaculum fili* was sufficient to enhance colitis in non-drinking mice. Our study provides a model to study binge drinking-microbiota interactions and their effects on host disease and reinforces the pathogenic function of *Allobaculum* spp. as colitogenic bacteria. Our findings illustrate how chronic binge drinking-induced alterations of the microbiome may affect susceptibility to IBD onset or flares.

## Introduction

Alcoholism has been studied extensively as a risk factor for chronic diseases such as diseases of the digestive system, specifically liver diseases, neuropsychiatric disorders, and cardiovascular diseases.^1–6^ Alcohol consumption is associated with numerous alterations in intestinal defense, that contribute to the pathology of steatohepatitis and liver disease.^6,7^ Studies in preclinical models and people have shown that chronic ethanol consumption promotes intestinal permeability, increases oxidative stress and impairs epithelial function that contributes to the translocation of gut microbial products into circulation.^8–11^

Gastrointestinal bacteria and metabolites are also affected in alcoholism.^12–14^ Previous studies in both humans and animal models of alcoholism showed intestinal bacterial overgrowth and fecal microbiome dysbiosis.^5,6,15–19^ Additionally, treatment with antibiotics reduce intestinal and liver injury supporting a role for the microbes in alcohol-induced pathogenesis.^20,21^

While alcohol-derived intestinal alterations, *e.g.*, epithelial barrier leak and inflammatory immune cell changes, are reminiscent of inflammatory bowel diseases (IBD) pathology, the contributions of chronic binge ethanol exposure for intestinal diseases outside of the alcoholic liver disease context is understudied. Although a prospective cohort study found no association between alcohol use and the risk of developing IBD, a few studies have suggested that consumption of alcohol can worsen gastrointestinal symptoms in IBD patients.^22–26^ Most preclinical animal studies of chronic alcohol consumption employ either a model of intragastric continuous ethanol feeding or the chronic and binge ethanol feeding model (NIAAA model)^27^ that results in hepatic injury and steatosis. As such, both these ethanol exposure methods are problematic for a refined understanding of intestinal specific responses. Moreover, the NIAAA model uses the Lieber-DeCarli diet, an ethanol-containing high-fat liquid chow provided *ad libitum*,^28^ which contrasts with the binge drinking behavior mostly observed in humans, particularly in adolescent age groups^4,29^ and adds the confounder of a high-fat liquid diet.

To carefully investigate the direct effects of binge drinking on the colonic mucosa and gut microbiome, we sought to develop a preclinical model of binge drinking that (i) mimicked human consumption, (ii) did not involve a dysbiosis-promoting or liver-injuring high fat diet, (iii) preserved standard laboratory mouse water and chow consumption behavior, and (iv) did not induce liver damage. We also leveraged gnotobiotic husbandry techniques to specifically interrogate the effect of chronic binge drinking on the microbiota. This model and approaches enabled us to both uncover that ethanol drinking effects persist beyond the drinking period and to identify *Allobaculum spp*. as an ethanol drinking-associated bacterium that potentiates colitis.

## Results

### Characterization of a chronic binge ethanol drinking preclinical model in mice

**Figure 1.**
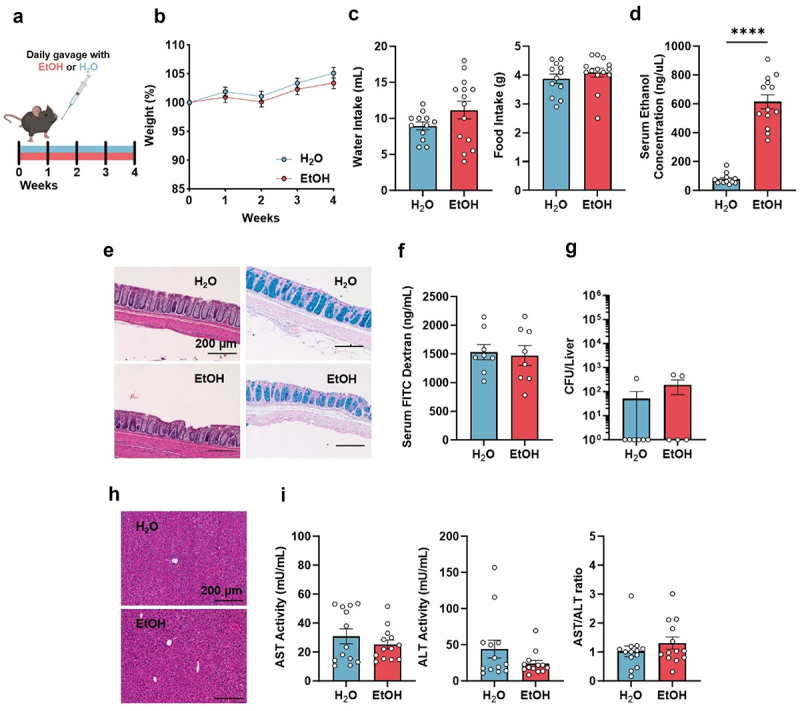
A model of chronic binge drinking.

To examine the effects of chronic binge ethanol consumption on intestinal inflammatory diseases and disentangle such effects from the gut-liver axis reported in models of severe alcoholism,^7,8,10,20,23,24^ we designed a model of binge drinking. We orally gavaged mice daily for 4 weeks with 3 mg of ethanol per gram mouse weight (EtOH) or an equivalent volume of water as control (H_2_O) (Figure 1(a)), while allowing *ad libitum* access to standard mouse chow and drinking water. There were no differences in body condition measured by body weight (Figure 1(b)), nor in food or water intake (Figure 1(c)), despite daily overt manifestations of drunkenness and increased serum ethanol levels after drinking (Figure 1(d), Videos 1–4). These levels recapitulate the NIAAA definition of binge drinking in humans (blood alcohol concentration − 800 mg/l).^29^ Although previous studies have reported colonic epithelial defects using other *in vitro* and *in vivo* models of alcoholism,^9–11,19^ we did not observe signs of direct tissue damage caused by our model of chronic binge drinking, as histological analysis of these binge drinking mice showed similar colon tissue architecture with no signs of colonic injury, inflammation or mucin-producing goblet cell depletion as assessed by review of both H&E and Alcian blue-stained colonic sections (Figure 1(e) and Supplementary Table S1). We also examined epithelial barrier function using two approaches, a serum fluorescence measurement following low molecular weight FITC-dextran gavage and bacterial culture of the liver. We found no differences between our ethanol-exposed and control groups (Figure 1(f,g)). Importantly, and contrary to previously published models of chronic ethanol consumption, we did not observe any evidence of liver damage in our model of chronic binge drinking, as assessed by histological analysis for the presence of steatosis, necrosis, portal or lobular inflammation; AST and ALT transaminase activity in the serum or increased serum AST/ALT ratio, which is associated with alcohol-related liver disease (Figure 1(h,i)). These results suggest that our proposed model of chronic binge drinking can be used to investigate effects of alcohol consumption in intestinal diseases, particularly the colon and its microbiota, without the confounding effect of severe tissue damage, alcoholic-liver disease, or marked dietary changes.

(a). Scheme of ethanol administration. Mice were gavaged daily for 4 weeks with 3 mg EtOH per gram mouse body weight or an equal volume of water as a control (H_2_O). (b). Percentage weight gain. H_2_O *n* = 23; EtOH *n* = 22. (c). Daily water (left) and food (right) intake. H_2_O *n* = 12; EtOH *n* = 14. (d). Serum EtOH concentration 30 minutes after gavage. H_2_O *n* = 13; EtOH *n* = 13. (e). Colon histology 4 weeks after daily gavage with EtOH or H_2_O. Left panels: H&E staining, photomicrograph from one representative mouse per group; Right panels: Alcian Blue staining, photomicrograph from one representative mouse per group. (f). Barrier leak assay, serum FITC-dextran concentration 3 h after administration. H_2_O *n* = 8; EtOH *n* = 8. (g). Gut barrier translocation assay, CFU per liver in mice, H_2_O *n* = 7; EtOH *n* = 5. (h). Liver histology, representative photomicrographs from one mouse per group are shown. (i). Serum AST (left), ALT (middle) activity and AST/ALT ratio (right) in mice gavaged daily for 4 weeks, H_2_O *n* = 13; EtOH *n* = 13. Symbols represent data from individual mice or mouse samples from 1 (g), 2 (c, d, f, i) or 4 (b) individual experiments.

**Figure 2.**
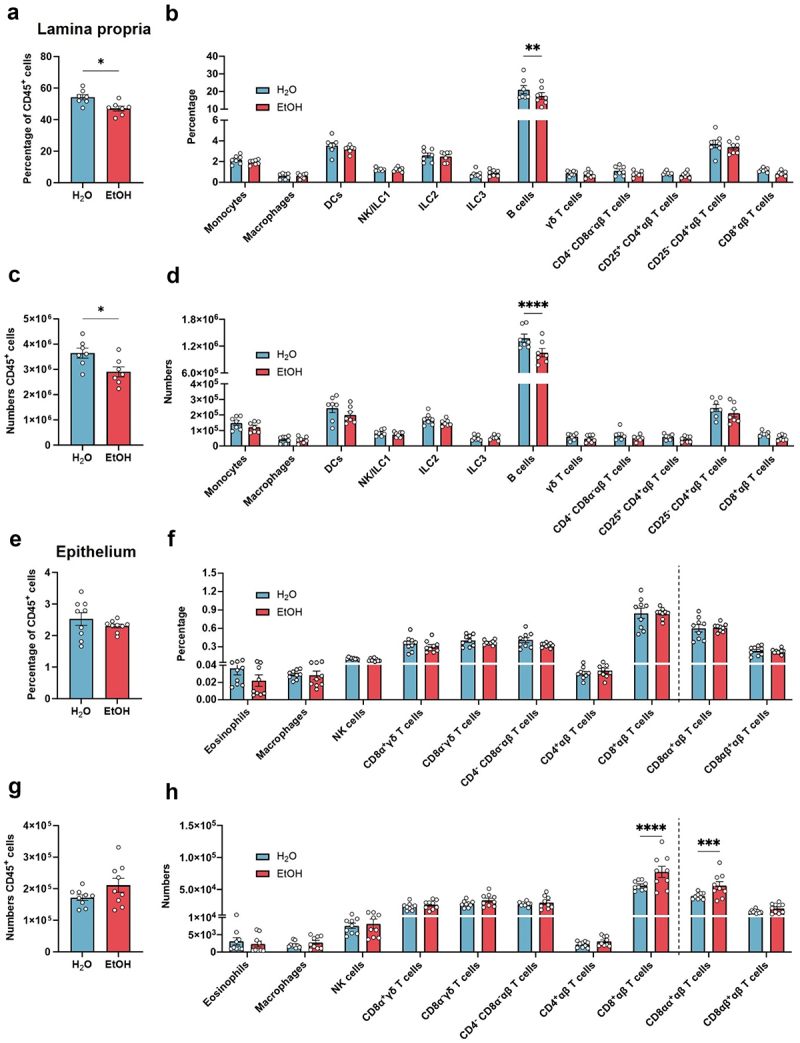
Cellular characterization of the chronic ethanol consumption model reveals subtle alterations in colon immune cell populations.

Prior studies of chronic heavy alcohol consumption support its effect on immunity, with alterations in immune gene expression, imbalances in the proportion of different immune cell populations, immune activation, production of cytokines and chemokines, and reduced capacity to fight infections.^7,30–36^ We examined if the chronic ethanol consumption employed in our model caused any alterations in the colonic immune system. In the colon lamina propria, apart from a modest but significant reduction in both the percentage and numbers of CD19^+^ B cells, we found that the remaining immune cell populations analyzed were otherwise similar between ethanol drinking and control mice (Figure 2(a–d)). The colon epithelium, which acts as the first layer of protection against intestinal pathogens, has specialized resident immune cells, particularly intraepithelial lymphocytes, that provide both pro-homeostatic and immunostimulatory signals to epithelial cells.^37–39^ Therefore, we analyzed the immune cells present in the epithelial layer of the EtOH drinking and control mice. We found an increase in CD8^+^ αβ T cells numbers, particularly in the gut-specific subpopulation that expresses the homodimer CD8αα, while the percentage and numbers of the remaining immune populations remained unchanged (Figure 2(e–h)). Previous studies showed that gut intraepithelial lymphocytes secrete cytokines, in particular IFNγ, that are not only critical for intestinal defense against pathogens and cancer, but also crucial for regulation of intestinal homeostasis.^40^ However, the amount of IFNγ-producing immune cells was not altered by ethanol consumption as compared to controls (Figure S1). Our results suggest that chronic binge drinking, in and of itself, does not cause major immune defects that would result in inflammation or tissue damage in the colon.

Percentage (a, b) and numbers (c, d) of total CD45^+^ immune cells and distinct immune cell populations in colon lamina propria. H_2_O *n* = 7; EtOH *n* = 7. Percentage (e, f) and numbers (g, h) of total CD45^+^ immune cells and distinct immune cell populations in colon epithelium. H_2_O *n* = 9; EtOH *n* = 9. Symbols represent data from individual mice from 2 independent experiments.

### Chronic ethanol binge drinking affects epithelial turnover

**Figure 3.**
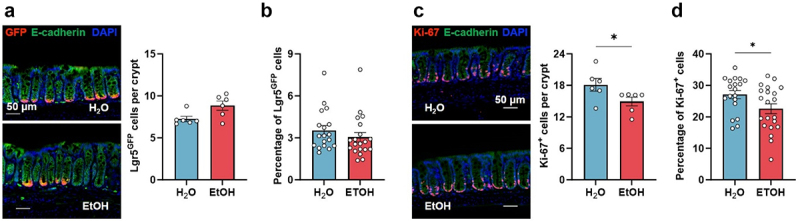
Binge ethanol consumption affects colonic epithelial cell turnover.

Previous studies using different models of alcoholism have shown that chronic ethanol drinking affects epithelial barrier permeability, with alterations in tight junction proteins and in intestinal epithelial cell proliferation.^9–11,41^ As we did not observe any alterations of the epithelial barrier or its permeability (Figure 1(e–g)), we next investigated if the colonic epithelium, in particular the proportion of colonic epithelial stem cells and epithelial proliferation, were affected in our model of chronic binge drinking. Despite observing similar numbers of LGR5^+^ epithelial stem cells by immunofluorescence micr and flow cytometry (Figure 3(a,b)), we found an imbalance of epithelial cell turnover with a small yet statistically significant reduction in the proportion of Ki-67^+^ proliferating cells in the ethanol-drinking versus control group (Figure 3(c,d)), when measured, respectively, as numbers of LGR5^+^ or Ki-67^+^ cells per crypt. We confirmed that the cellular composition of the colon epithelium was not altered by chronic binge ethanol drinking when compared to controls by assessing the expression of genes associated with the development or function of the intestinal epithelium (*Krt20*) and of specific cell populations: epithelial stem cells (*Lgr5*, *EphB2*), absorptive enterocytes (*Vil1*), enteroendocrine cells (*Chga*), tuft cells (*Dclk1*, *Trpm5*) and goblet cells (*Klf4*, *Tff*, *Fut2*, *Muc* genes) (Figure S2(a)).

(a). Quantification of GFP^+^ colon epithelial stem cells per crypt. Left: representative immunofluorescence micrographs from one mouse per group; Right: cell counting quantifications, H_2_O *n* = 6; EtOH *n* = 6. (b). Flow cytometry analysis of the percentage of GFP^+^ colon epithelial stem cells, H_2_O *n* = 18; EtOH *n* = 20. (c). Quantification of Ki67^+^ colon epithelial cells per crypt. Left: representative immunofluorescence micrographs from one mouse per group; Right: cell counting quantifications. H_2_O *n* = 6; EtOH *n* = 6. (d). Flow cytometry analysis of colonic epithelial cells (CECs), percentage of Ki67^+^ CECs shown. H_2_O *n* = 19; EtOH *n* = 21. Symbols represent the mean epithelial crypt counts (between 4 and 13 crypts counted, determined by crypt orientation) per mouse from 2 independent experiments (a, c) or data from individual mice from 3 independent experiments (b, d).

While most consumed alcohol is metabolized in the liver; alcohol metabolism can also occur in the peripheral tissues. Thus, we next investigated if ethanol metabolism in the colonic epithelium was altered by chronic binge drinking by investigating the expression of members of the alcohol (*Adh*) and aldehyde (*Aldh*) dehydrogenases enzyme family required to convert ethanol to acetate. We found that ethanol drinking and control mice express these genes in similar amounts (Figure S2(b)), supporting that ethanol metabolism in the colonic epithelium is not affected by our chronic binge drinking intervention.

### Increased sensitivity to intestinal disease in chronic binge ethanol drinking mice

**Figure 4.**
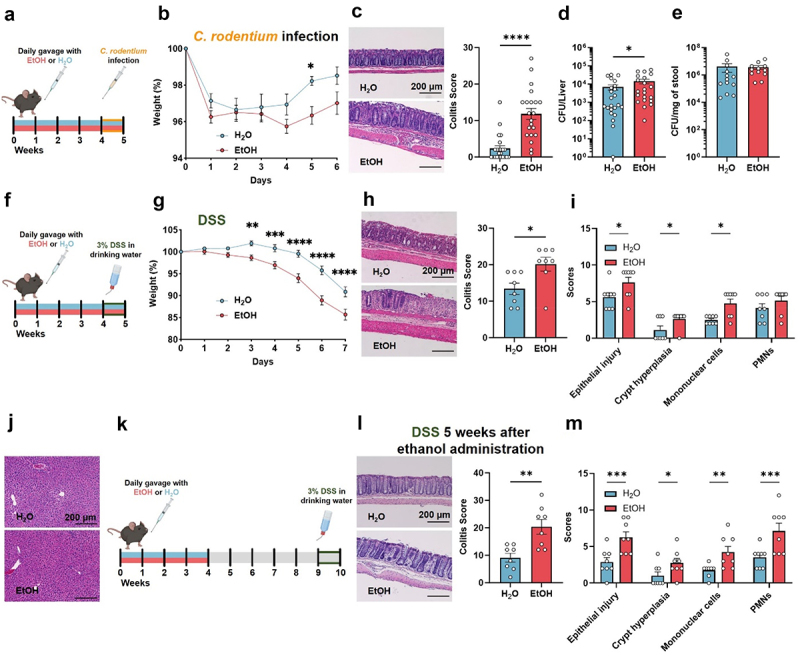
Binge ethanol drinking increases susceptibility to intestinal infection and dextran sulfate sodium-induced colonic injury and inflammation.

Our results so far present a model of chronic binge drinking with observable daily intoxication but with only subtle alterations in intestinal homeostasis and defense and no direct intestinal damage. However, several previous studies of alcohol consumption and alcoholism in animal models and humans have reported intestinal barrier dysfunction, intestinal inflammation, microbial dysbiosis, and increased gastrointestinal infection susceptibility in alcoholics.^5,6,8,9,13,17,19,20,42^ Recently, Martino and colleagues^43^ demonstrated that ethanol-derived acetate can reprogram the gut microbiota in a manner comparable to drinking and independently of liver injury. Therefore, we wondered if chronic binge ethanol drinking could promote intestinal inflammatory diseases in response to a second pathogenic event. To investigate this idea, we infected mice after 4 weeks of chronic binge ethanol drinking with an attenuated strain of *Citrobacter rodentium*, a rodent pathogen that mimics attaching-and-effacing pathogenic *E. coli* infections in humans (Figure 4(a)). Although the initial weight loss after infection was the same between groups, over the course of the experiment, we found an increased weight loss and slower recovery in ethanol drinking mice compared to controls, resulting in a 5 times higher colitis score (Figure 4(b,c)). In accordance with that observation, we detected increased bacterial translocation to the liver of mice in the ethanol group, suggesting an increase in intestinal barrier breach by this pathogen, while total bacterial load in the stool remained unchanged (Figure 4(d,e)).

(a). Mice were gavaged daily for 4 weeks with 3 mg EtOH per gram mouse body weight (EtOH) or an equal volume of water as a control (H_2_O) followed by a single oral administration of *Citrobacter rodentium*. (b). Percentage weight loss, H_2_O *n* = 23; EtOH *n* = 21. (c). Histological analysis of colons, representative photomicrographs using H&E staining and histology-based colitis score, H_2_O *n* = 23; EtOH *n* = 21. (d). *C. rodentium* CFU per liver, H_2_O *n* = 23; EtOH *n* = 21. (e). *C. rodentium* CFU per mg stool, H_2_O *n* = 13; EtOH *n* = 12. (f). Mice were gavaged daily for 4 weeks with 3 mg of ethanol per gram mouse body weight (EtOH) or an equivalent volume of water as a control (H_2_O) followed by 3% (w/v) of dextran sulfate sodium (DSS) in the drinking water for 5 days and then 2 days of normal drinking water. (g). Percentage weight loss, H_2_O *n* = 17; EtOH *n* = 16. (h, i) Histological analysis of colons, representative micrographs using H&E staining and histology-based colitis score and sub-scores categories, H_2_O *n* = 8; EtOH *n* = 8. (j). Liver histology after DSS, representative photomicrographs from each group are shown. (k). Mice were gavaged daily for 4 weeks with 3 mg EtOH per gram mouse body weight (EtOH) or equal volume of water as a control (H_2_O) for 4 weeks, given an abstinence period of 5 weeks followed by DSS in the drinking water as above. (l, m). Analysis of colon histology using H&E staining, histology-based colitis scores and sub-score categories, H_2_O *n* = 8; EtOH *n* = 8. Symbols represent data from individual mice or mouse sample from 2 (e, h, i, l, m), 3 (b, c, d) or 4 (g) independent experiments.

While there is no consensus on the contribution of alcohol consumption as a risk factor for the development of IBD, alcoholism has been associated with dysbiosis similar to IBD, and worsening of symptoms has been reported in a cohort of IBD patients after ethanol consumption.^15,23–25^ To address this knowledge gap, we examined the effect of chronic binge ethanol drinking in a model of chemically induced colonic injury and inflammation using Dextran Sulfate Sodium salt (DSS) in the drinking water (Figure 4(f)). Mice in the ethanol group lost more weight than controls upon DSS treatment (Figure 4(g)). Although no significant differences between groups were observed in immune cell numbers or production of IFNγ, IL-17A and IL-22 in the lamina propria when analyzed by flow cytometry (Figure S3), histological analysis revealed increased colitis scores in the ethanol group, with an overall increase in the majority of the disease parameters analyzed (epithelial injury, epithelial crypt hyperplasia, and mononuclear cell infiltration; weighted for the extent of disease) (Figure 4(h,i)). No liver damage was observed (Figure 4(j)), supporting that the alcohol-dependent potentiation of colonic injury and inflammation occurs in the absence of liver injury.

We next questioned if the effect of chronic binge ethanol drinking on colitis required active drinking behavior or if ethanol consumption had a prolonged effect on the susceptibility to colitis. To address that question, we separated the month-long chronic binge ethanol drinking intervention from the colitis induction by 5 weeks (Figure 4(k)). After five weeks of abstinence from ethanol, ex-drinker mice had worse colitis as compared to control mice across all parameters analyzed (Figure 4(l,m)), demonstrating that the effects of chronic binge drinking in promoting intestinal injury and inflammation persist beyond the active ethanol consumption period.

### Microbiota is required and explains the chronic drinking-derived sensitivity to colitis

**Figure 5.**
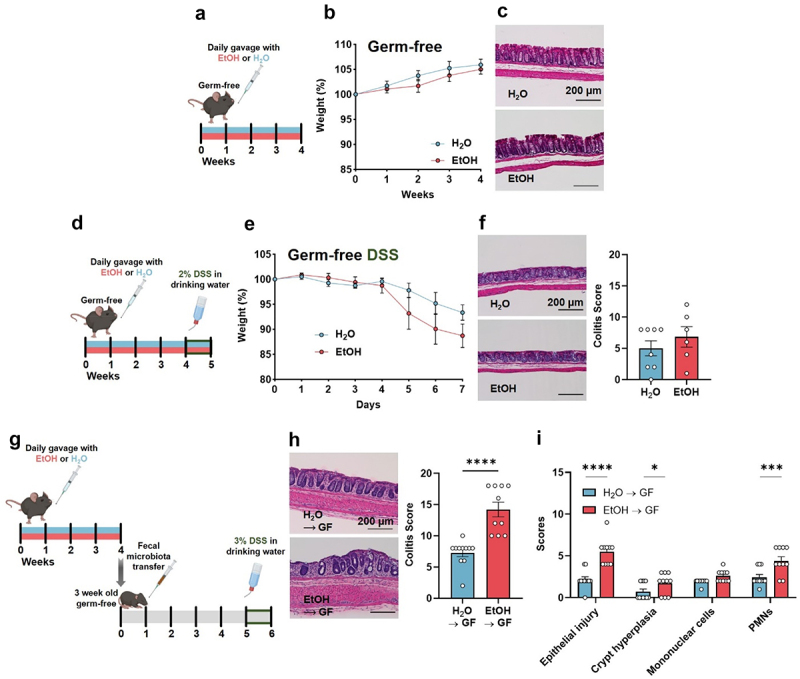
Susceptibility to colonic injury and inflammation in binge ethanol drinking mice is microbiota-dependent and transferable by cecal microbiota transplantation to ethanol-naïve mice.

Alcoholism has been associated with dysbiotic alterations in the gut microbiome.^5,13,17,20^ Having observed that (i) the effect of chronic binge drinking on susceptibility to colonic inflammation could be dissociated in time from the act of drinking and (ii) in the absence of an inflammatory trigger that mice did not display observable drinking-related intestinal damage, we hypothesized that the colon microbiota in ethanol consuming mice could explain their predisposition to intestinal injury. To study this, we first used germ-free (GF) mice subjected to the same chronic binge ethanol drinking intervention (Figure 5(a)). In accordance with what we observed with conventional mice, GF ethanol consuming mice had similar weight gain to water controls and no intestinal pathology (Figure 5(b,c) and Supplementary Table S2). Afterwards, we wondered if chronic binge ethanol drinking mice would still be prone to worse colitis in the absence of a microbiota (Figure 5(d)). Contrary to observations in conventional mice, GF alcohol drinking mice displayed similar colitis score to controls (Figure 5(e,f)). These results indicate that the chronic binge ethanol drinking derived susceptibility to colitis requires the presence of a gut microbiota. Subsequently, we investigated if alterations in the microbiota caused by chronic drinking were sufficient to explain the increased colitis in ethanol drinking mice using a cecal microbiota transplantation (CMT) approach (Figure 5(g)). We collected the cecal contents of mice under the chronic binge ethanol drinking intervention and that of controls at the terminal timepoint and transferred these samples into 3 weeks old GF mice. Recipient mice aged for 5 weeks post CMT (8 weeks of age), before colitis induction with DSS. Mice that never consumed ethanol but received the microbiota from ethanol consuming mice had increased colitis scores when compared to mice that received the microbiota from control mice (Figure 5(h,i)). These results support that specific microbiome alterations in chronic binge ethanol drinking mice are transferable by CMT and are responsible for increased sensitivity to colonic injury and inflammation with DSS.

(a). Germ-free (GF) mice were gavaged daily for 4 weeks with 3 mg EtOH per gram mouse body weight (EtOH) or an equal volume of water as a control (H_2_O). (b). Percentage weight gain, H_2_O *n* = 23; EtOH *n* = 21. (c). Analysis of colon histology using H&E stained sections. Representative photomicrographs are shown for GF mice. (d). GF mice were gavaged daily for 4 weeks with 3 mg EtOH per gram mouse body weight (EtOH) or water as a control (H_2_O) followed by DSS in the drinking water. (e). Percentage weight loss, H_2_O *n* = 8; EtOH *n* = 6. (f). Analysis of colon histology using H&E stained sections, representative photomicrographs and histology-based colitis score are shown for EtOH-consuming GF mice and controls after DSS, H_2_O *n* = 8; EtOH *n* = 6. (g). Mice were gavaged daily with 3 mg EtOH per gram mouse body weight (EtOH) or water as a control (H_2_O) for 4 weeks and their cecal contents were harvested. The cecal contents of EtOH mice or water controls were then transferred to 3 week old GF mice by gavage. At 8 weeks of age, recipient mice (EtOH➙GF or H_2_O➙GF) were treated with DSS. (h, i). Analysis of colon histology using H&E stained sections, representative photomicrographs, colitis scores and sub-scores categories, H_2_O *n* = 11; EtOH *n* = 10. Symbols represent data from individual mice from 1 (h, i), 2 (e, f) or 4 (b) independent experiments.

### *Binge ethanol drinking-dependent* Allobaculum sp. *blooms exacerbate DSS-induced intestinal injury and inflammation*

**Figure 6.**
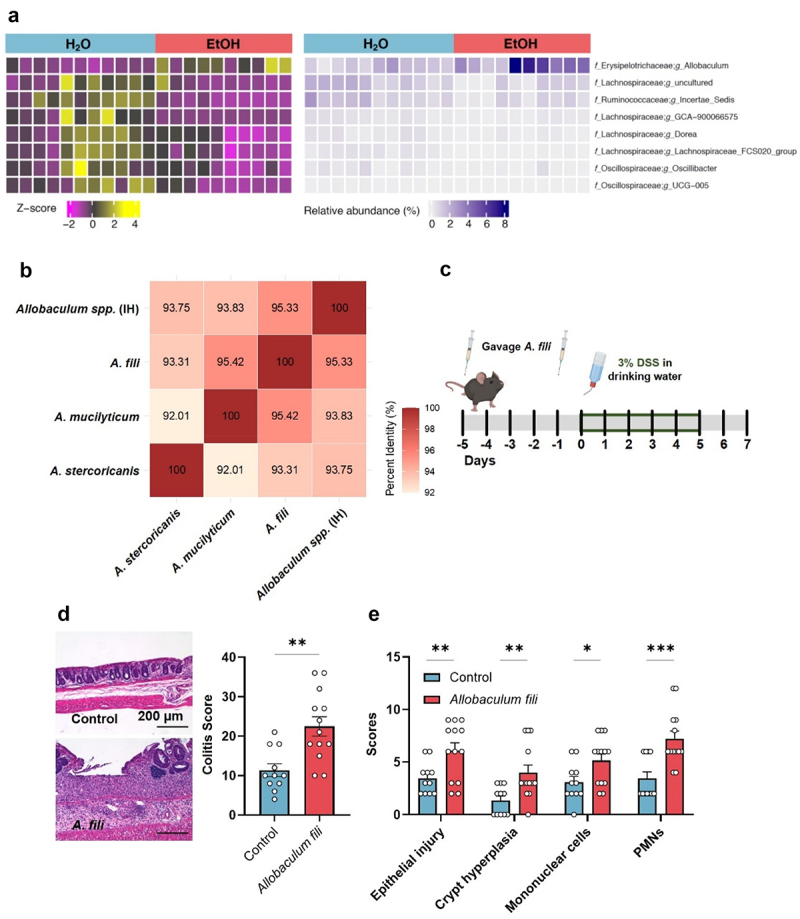
Binge ethanol-driven *Allobaculum sp*. blooms exacerbate dss-induced colonic injury and inflammation.

After finding that factors in the microbiome of ethanol-consuming mice result in increased colitis, we performed 16S rRNA gene amplicon sequencing analysis on the cecal contents of CMT recipient mice after colitis induction. Differential taxon abundance analysis using MaAslin2^44^ indicated that 8 taxa were differentially abundant between chronic binge ethanol drinking mice and controls. Notably, whereas most of the differentially abundant taxa had low abundance and were reduced in drinking mice, we found that *Allobaculum sp*. was the only taxon increased in ethanol consuming mice (Figure 6(a), Supplementary Table S3). *Allobaculum* is a genus of Gram-positive, strictly anaerobic bacteria.^45^ In parallel, we examined the cecal microbiomes of chronic binge drinking and control mice without DSS colitis induction. Notably, while there were no statistically different differences associated with the ethanol exposure, there was a trend for increased *Allobaculum sp* abundance in binge drinking mice after 4 weeks of ethanol exposure (Supplementary Table S4).

(a). 16S rRNA gene amplicon sequencing of cecal contents from mice subjected to cecal microbiota transfer and treated with DSS. Heatmaps with Z-score values (left) and relative abundance (right) for differentially abundant bacterial genera between binge ethanol drinking mice (EtOH) and controls (H_2_O). (b). Identity matrix of *Allobaculum spp*., designated in-house (IH), constructed from partially assembled and sequenced 16S rRNA genes and analyzed against known species of the *Allobaculum* genus. (c). Mice were gavaged twice with *Allobaculum fili* or control followed by DSS in the drinking water. (d, e). Analysis of colon histology using H&E stained sections, histology-based colitis scores and sub-scores categories in mice gavaged with *A. fili* or PBS control. H_2_O *n* = 11; EtOH *n* = 13. Symbols represent data from individual mice from 2 independent experiments.

*Allobaculum* species are routinely detected in 16S rRNA gene amplicon sequencing analyses of human and mouse fecal microbiomes, and recently two new species, *Allobaculum mucilyticum* and *Allobaculum fili*, were isolated from the feces of IBD patients.^46,47^ To interrogate the phylogenetic relationship between the *Allobaculum sp*. observed in our mice and previously described species, we designed specific primers to the *Allobaculum sp*. 16S rRNA gene to assemble the in-house *Allobaculum sp*. 16S rRNA gene sequence. We constructed an identity similarity matrix to *Allobaculum stercoricanis*, *Allobaculum mucilyticum* and *Allobaculum fili* using ClustalOmega (Figure 6(b)).^48,49^ We found that despite not reaching species-level similarity, the in-house *Allobaculum sp*. was most closely related to *Allobaculum fili*. Following the finding that *Allobaculum* was the only taxon increased in the ethanol drinking microbiome, we questioned if the increase in *Allobaculum sp*. could explain the increased susceptibility to colonic injury and inflammation. As we encountered technical challenges in isolating the in-house *Allobaculum sp*. in mono-culture, we employed *Allobaculum fili* as the closest culturable *Allobaculum sp*. To study *Allobaculum*’s influence on susceptibility to colitis, we orally gavaged conventional mice with *Allobaculum fili* before inducing colitis (Figure 6(c)). Strikingly, we found that mice that received *Allobaculum fili* developed more severe colitis across all parameters, when compared to control mice (Figure 6(d,e)). These findings support that *Allobaculum spp*. that increase upon chronic binger ethanol drinking are colitogenic and can contribute to intestinal pathology.

## Discussion

Most animal models of chronic alcohol consumption have documented gut microbiome changes, but they also administer ethanol *ad libitum* in either food or drinking water, which poorly models human binge drinking behavior.^5,13,17,20^ In this study, we employed a strategy of daily chronic binge ethanol drinking with observable and measurable alcohol intoxication. This approach revealed only minor alterations in the immune cells and epithelial barrier, but a heightened susceptibility to infection and colitis with an increase in an *Allobaculum sp*. in ethanol consuming mice. In contrast with previous studies with documented liver injury,^8–11^ we did not find strong defects in epithelial barrier function and permeability cause by ethanol. However, we did observe increased permeability and bacterial translocation to the liver in ethanol consuming mice upon infection. The microbiome alterations observed in this study also contrast with the broader changes in previous studies of alcoholism and alcoholic liver disease, *e.g*., we do not detect expansion of opportunistic pathogenic *Proteobacteria*.^12,18–20,50^ While these changes might be explained by differences in ethanol administration and disease status, they might also reflect a limitation of the usage of laboratory animals, as fecal *Proteobacteria* are not usually observed members of conventional laboratory mouse fecal microbiota in many vivaria.^51^ Furthermore, additional signals, such as circulating inflammatory cues or feedback from liver damage,^7,42^ might be required to trigger the *in vivo* ethanol effects observed in epithelial cells in some models.

A large European international study found no direct correlation between lifetime alcohol consumption and the development of both ulcerative colitis and Crohn’s disease,^22^ which was reaffirmed by a meta-analysis of 16 epidemiological studies.^52^ However, alcohol consumption in IBD patients was reported to increase the risk of relapse,^26^ worsen disease symptoms upon a flare^23^ and increased susceptibility to intestinal infections, which the authors later confirmed in mice using a model of acute binge drinking after DSS-induced colitis.^53^

Alcohol-induced intestinal dysbiosis and inflammation share remarkable similarities with IBD.^7,23–25^ The expansion of *Allobaculum sp*. in chronic ethanol drinking mice observed in our study links ethanol consumption and IBD with potential clinical implications. Interestingly, in a previous study from our laboratory examining the microbiota of mice carrying the Crohn’s disease risk associated polymorphism *ATG16L1* T300A, we found that *Allobaculum* abundance, among other taxa, was increased in mice bearing the IBD genetic risk allele when comparted to wild type mice.^54^ Two *Allobaculum spp*., *Allobaculum mucilyticum* and *Allobaculum fili,* isolated from IBD patients using bacterial reactive fecal immunoglobulin A are colitogenic in mouse models, and these findings enabled investigators to better understand how bacterial-bacterial interactions influence intestinal epithelial response and shape mucosal immunity.^46,47,55^ Specifically, the exact composition of the gut microbiota and the interactions between commensal species are critical for this pro-inflammatory effect, as *Allobaculum mucilyticum*’s colitogenic action is counteracted by the immunomodulatory effects of *Akkermansia muciniphila*.^55^ *Allobaculum mucilyticum* is also a potent carbohydrate digestor and mucin degrader^56,57^ that exacerbates colitis in mouse models with an otherwise non-colitogenic simplified microbial community.^55^

Herein, we demonstrate that a previously uncharacterized *Allobaculum sp*. increases upon chronic ethanol consumption and that *Allobaculum fili*, a culturable and close relative, also aggravates colonic injury and inflammation. These data suggest that the effects of chronic ethanol drinking in the development of IBD might be dependent on the host microbiota and expansion of potential opportunistic bacteria. Members of the *Allobaculum* genus are emerging as examples of bacteria that, although present in in healthy individuals,^58^ rapidly change in response to environmental alterations such as diet variations and obesity,^59–61^ chronic ethanol consumption (this study) and alcoholic liver disease,^20^ and inflammation-derived colorectal cancer.^62^

Chronic ethanol consumption has been shown to profoundly alter the metabolome of the distal gastrointestinal tract, *e.g*., increased fatty acid and steroids and decreased amino acids and branched chain amino acids, and short chain fatty acids, with the exception of acetate.^14^ Although these changes may originate from differences in microbiota metabolism, a recent study showed that direct ethanol metabolism is almost entirely carried out by the host.^43^ Additionally, the same study showed that host derived acetate originated from ethanol degradation was sufficient to cause the microbiome alterations observed with ethanol feeding.

Notably, *Allobaculum spp*. can produce ethanol as well as the immunomodulatory metabolites lactate and butyrate.^45,46^ Additionally, *Allobaculum mucilyticum* and *Allobaculum fili*, but not *Allobaculum stercoricanis*, are weak metabolizers with respect to the IBD-biologically relevant processes of nitrate reduction and indole production.^46^ Similar to *Allobaculum mucilyticum*, the carbohydrate degradation enzymes produced by *Allobaculum fili*, such as β-Galactosidase, β-Glucuronidase and N-Acetyl-β-glucosaminidase, can degrade the protective intestinal mucin barrier and exacerbate intestinal inflammation.^46,56,57^ Whether members of the *Allobaculum* genus, in particular *Allobaculum fili* investigated here, promote a colitogenic environment only by degrading mucins or if other metabolic processes and metabolites, such as local production of ethanol, are also involved is an important question that requires further studies, particularly in the absence, so far, of detectable canonical virulence factors.

The gut microbiota has also been investigated for its role in the neurological and behavioral alterations observed in alcohol use disorder.^3,4,63^ Two studies in subjects diagnosed with alcohol dependence showed correlations between intestinal permeability, inflammatory markers, gut dysbiosis and microbial products, and behavioral markers of alcohol dependency.^35,64,65^ Recently, studies using mouse models and a cohort of young binge drinkers established a link between gut microbiota alterations and changes in brain function and behavior. The authors identified microbiota and microbial metabolic modules that are altered in young high binge drinkers and found both positive and negative associations between specific bacterial taxa and alcohol craving, emotion recognition and impulsivity.^4^ In a separate study that employed mice and fecal microbiome transplantation from alcohol-dependent (AD) patients, mice that received stool from AD patients showed decreased social behavior, more depression-like behavior and altered brain function with reduction in the excitatory neurotransmitter glutamate.^63^ In AD-colonized mice, microbial ethanol production resulted in metabolism changes with reduction in ketogenesis and β-hydroxybutyrate and that β-hydroxybutyrate positively correlate with glutamate levels and sociability index. As noted above, members of the *Allobaculum* genus can also produce ethanol.^45,46^ Furthermore, transcriptomic profiling following alcohol consumption in mice identified several differential expressed intestinal genes, one of which is prokineticin 2 (*Prok2*).^36^ Prokineticin 2 has pleiotropic effects and has been associated with IBD, neuronal survival and pain sensation.^66–70^ Surprisingly, the authors found that, among others, *Allobaculum* was negatively associated with *Prok2*, hinting at the possibility that *Allobaculum spp*. also bridges alcohol consumption and neuronal function.

In summary, we have developed a model of daily binge ethanol drinking that circumvents the confounding factors of liver injury, direct colon tissue damage and drastic dietary perturbations and can be utilized to investigate the impact of binge ethanol drinking on organ physiology and disease. This model might be particularly useful to interrogate the alcohol-microbiota axis in neuronal and endocrine disorders, which are also affected by alcohol consumption similar to IBD. Our study shines a light on *Allobaculum*’s potential role in alcohol-triggered IBD flares and symptom exacerbations; and further investigations are required to reveal its microbial products and interactions with other members of the microbiota that influence gut homeostasis.

## Materials and methods

### Mice husbandry

C57BL/6J and Lgr5-eGFP-creERT2 (Lgr5^GFP^) mice were obtained from The Jackson Laboratory, bred and housed in microisolator cages in the barrier facility at the Harvard T.H. Chan School of Public Health. Germ-free C57BL/6J mice were bred and maintained in semi-rigid gnotobiotic isolators in the Harvard T. H. Chan Gnotobiotic Center for Mechanistic Microbiome Studies. Experimental male and female mice were above 8 weeks of age. Germ-free mice receiving cecal microbiota transplantation (CMT) were 3 weeks old and were housed in individually ventilated cages after CMT. Mice were single housed for 1–2 days to determine daily food and water intake. Animal studies and experiments were approved and carried out in accordance with Harvard Medical School’s Standing Committee on Animals and the National Institutes of Health guidelines for animal use and care.

### Ethanol administration

Mice were gavaged daily with 3 mg of ethanol per gram of mouse body weight of a 20% ethanol solution for the time indicated in the figures and figure legends. Body weight was measured weekly.

### Bacterial infection, colonization and cecal microbiota transplantation (CMT)

*Citrobater rodentium* (DBS100 strain) was generously provided by Dr. John Leong (Tufts University School of Medicine). Mice were orally infected with approximately 4 × 10^9^ CFU of *C. rodentium*, as previously described.^71^ Briefly, *C. rodentium* was inoculated in 15 mL of Difco LB broth, Miller (BD Bioscience) in 50 mL tubes and cultured overnight in a 37°C shaking incubator. *C. rodentium* culture was centrifuged, resuspended in 1.5 mL of PBS and 200 μL of suspension was gavaged per mouse. Body weight was measured daily. Colony-forming units of the initial suspension, stool and homogenized livers were determined by serial dilution and plating in Difco LB agar, Miller (BD Bioscience) plates. *Allobaculum fili* gavage suspension was prepared in a similar way, but *A. fili* was cultivated for 48 hours under anaerobic conditions at 37°C in enriched Gut Microbiota Medium.^46,72^ For CMT, fresh cecal contents were resuspended in PBS under anaerobic conditions and gavaged into germ-free mice as a 10% (w/v) suspension.

### Dextran sulfate sodium salt (DSS)-induced colitis model

Mice were treated with 3% (w/v) DSS (Thermo Scientific) in the drinking water for 5 days and followed by regular drinking water for 2 days. Germ-free animals were similarly treated with 2% (w/v) DSS. Body weight was measured daily, and mice were euthanized at day 7 post DSS administration.

### Serum AST and ALT activity, and ethanol concentration

Blood was collected into serum separator tubes, spun for 5 min at 5000 rpm and serum collected. Serum AST and ALT activity and ethanol concentrations were determined using an AST activity assay kit, ALT activity assay kit and ethanol assay kit, respectively (Sigma-Aldrich).

### Colonic barrier function testing with FITC-dextran

Mice were gavaged with a 100 mg/mL 4 kDa FITC-dextran (Sigma-Aldrich) solution in PBS (approximately 10 mg per 20 g mouse). Blood was collected 3 hours after gavage into serum separator tubes, spun for 5 minutes at 5000 rpm and serum collected. Serum samples were diluted 1:1 with PBS and fluorescence intensities measured with 485 nm excitation and 528 nm emission wavelength in a VERSAmax microplate reader (Molecular Devices).

### Histology and fluorescence microscopy

Liver lobes were separated, and colons were opened longitudinally, cleaned and fixed overnight in 4% paraformaldehyde (Sigma-Aldrich). Tissue paraffin embedding, sectioning and hematoxylin – eosin (H&E) staining was performed at the Harvard Medical School Rodent Histopathology Core. Alcian blue staining was performed to image goblet cells. Liver histology was scored blindly by a pathologist as absent (0), mild (1), moderate (2), or severe (3) for: steatosis; portal inflammation; lobular inflammation; necrosis. Colitis scores were determined by a pathologist who was blinded to the experimental conditions, as previously described.^73^ Briefly, the four histologic parameters were scored as absent (0), mild (1), moderate (2), or severe (3): mononuclear cell infiltration, polymorphonuclear cell infiltration (PMNs), epithelial crypt hyperplasia, and epithelial injury, and summed a cumulative colitis score. Cumulative colitis score was weighted in an extended colitis score for the percentage of tissue involvement in the disease using a multiplier: <10% (1); 10%–25% (2); 30%–50% (3); >50% (4) as follows: cumulative score * % involvement multiplier.

For immunofluorescence staining, sections were deparaffinized and rehydrated. Heat-mediated antigen retrieval was performed in Target Retrieval Solution, Citrate pH 6.1 (Agilent) for 20 minutes at 90°C. Slides were then washed in water and PBS and blocked with blocking buffer (PBS containing 3% BSA (Roche), 0.1% saponin (Sigma-Aldrich) and 0.1% Triton X-100 (Sigma-Aldrich)) with 3% donkey serum (Jackson ImmunoResearch), for 1 hour at room temperature. Primary antibodies rat anti-Ki-67 (1:300 dilution, 14-5698-82, ThermoFisher Scientific) and mouse anti-E-Cadherin (1:400 dilution, 36/E-Cadherin, BD Biosciences) were incubated in blocking buffer overnight at 4°C. Secondary antibodies AlexaFluor 594 donkey anti-rat IgG (1:300 dilution, 712-585-153, Jackson ImmunoResearch) and AlexaFluor 647 donkey anti-mouse IgG (1:300, ab150111, Abcam) were incubated 1.5 hours at room temperature. For GFP detection, goat anti-GFP antibody (1:200, ab6673, Abcam) and AlexaFluor 488 donkey anti-rat (1:300, 705-545-003, Jackson ImmunoResearch) were used as primary and secondary antibodies, respectively. Nuclear DNA was labeled with 0.5 μg/mL of DAPI. Images were acquired on a Nikon Eclipse microscope equipped with Nikon DS-Qi1Mc and Nikon DS-Fi2 cameras. Quantification of LGR5-GFP^+^ and KI-67^+^ cells was performed by counting the number of positive cells per individual crypt, 4 to 13 individual crypts were counted per experimental animal.

### Isolation of colonic lamina propria and epithelial layer

Colons were opened longitudinally, feces and debris gently removed, and washed in PBS, 2% FBS (Gibco), 5 mM HEPES (Gibco), 1 mM DTT (Sigma-Aldrich) for 10 minutes at 4°C. Cells in the epithelium were separated by incubating washed colon tissue twice in prewarmed PBS, 2% FBS, 5 mM HEPES, 5 mM EDTA (VWR) and rotated at 37°C for 15 and 10 minutes, respectively. Epithelial cell suspension was obtained by filtering with 100 μL filters. To isolate lamina propria cells, the remaining colon tissue was washed in PBS, cut into small pieces minced with scissors in a 1.5 mL tube with digestion media, and digested in 10 mL of RPMI containing 2% FBS, 5 mM HEPES, 1% penicillin/streptomycin (Corning), 0.5 mg/mL of collagenase D (Roche), 50 μg/ml DNase (Roche) and 0.5 units/ml Dispase II (StemCell Technologies) for 30 minutes at 37°C. Single cell suspension was obtaining by filtering the digested tissues with 70 filters.

### Flow cytometry

Fc receptor blocking with anti-CD16/CD32 (clone 93, Biolegend) was used in all experiments before surface and intracellular staining. Cell suspensions were stained with Aqua or near-IR LIVE/DEAD fixable viability dye for 10 minutes at room temperature followed by surface antibodies for 20 minutes at 4°C. For staining of intracellular cytokines, 10^6^ cells from the epithelial fraction suspensions were stimulated with Cell Stimulation Cocktail (plus protein transport inhibitors) (00-4975-03, eBioscience) in RPMI containing 10% FBS and 1% penicillin/streptomycin for 4 hours at 37°C. After stimulation, cells were Fc receptor blocked, surface stained, fixed with BD Cytofix for 30 minutes and permeabilized with BD Perm/Wash (BD Biosciences). Intracellular staining was performed in BD Perm/Wash using PE-Cy7 anti-mouse IFNγ (XMG1.2, BioLegend), PE anti-mouse IL-22 (1H8PWSR, eBioscience) or PerCP-Cy5.5 anti-mouse IL-17A (TC11-18H10.1, Biolegend) for 45 minutes at room temperature. For intracellular KI-67 staining in Lgr5^GFP^ mice, cells were first fixed with BD Cytofix for 20 minutes, followed by a second fixation step using eBioscience Foxp3/Transcription Factor Staining Buffer Set (Invitrogen) for 45 minutes. Intracellular staining with PE-Cy7 anti-mouse/rat Ki-67 (SolA15, eBioscience) was performed in permeabilization buffer for 45 minutes. Samples were acquired on an LSRII and analyzed with FlowJo (BD Biosciences). Immune cell populations analyzed and anti-mouse antibodies from BioLegend, eBioscience or BD Bioscience for surface staining are shown in Supplementary Table S5 and Supplementary Table S6, respectively.

### DNA/RNA isolation and real-time quantitative PCR

For RNA isolation from the colon epithelial fraction, cells were homogenized and snap frozen in Qiazol (Qiagen). RNA was extracted with chloroform following the manufacturer’s instructions and using Phase Lock Gel tubes (QuantaBio). cDNA was synthesized with High-Capacity RNA-to-cDNA™ Kit (Applied Biosystems) and quantitative real-time PCR was performed using KAPA SYBR FAST Universal Master Mix (KAPA Biosystems) on a Stratagene Mx3005P machine (Agilent Technologies). Primers used are listed in Supplementary Table S7. Gene expression was normalized to the housekeeping gene β-actin and presented using the 2^−ΔCt^ method. DNA isolation from bacterial cultures was performed using the QIAamp Fast DNA Stool Mini Kit (Qiagen). DNA from frozen stool samples of DSS-treated mice was extracted by the phenol-chloroform method after bead beating as previously described,^74^ with the exception of that a double 2.5 M LiCl precipitation step was included to clean up the DSS contamination.^75^

### 16S ribosomal RNA (rRNA) gene amplicon sequencing of mouse stool

The 16S rRNA gene amplification protocol was adapted from the Earth Microbiome Project.^76^ The 16S rRNA V4 region of extracted DNA was amplified by PCR and then purified by the AxyPrep Mag PCR Clean-Up Kit (MAG-PCR-CL-50, Corning) to remove free primers and primer dimers. The purified amplicon was quantified using a Qubit dsDNA HS assay (Q32854, Thermo Fisher) and equal amount (by mass) was pooled together. The 16S rRNA V4 library sequencing was performed on a MiSeq instrument (Illumina, San Diego, CA) using 150bp paired-end reading by the Molecular Biology Core Facilities (MBCF) at Dana-Farber Cancer institute (DFCI). The multiplexed raw sequencing data were imported to the QIIME2 environment (version 2020.8)^77^ and then low-quality bases were removed. The quality trimmed reads were joined, denoised, and checked for chimeras using DADA2 plug-in^78^ prior to taxonomic assignment. Taxonomic assignment of each amplicon sequence variant (ASV) was performed using a pre-trained Naive Bayes classifier with the SILVA database (version 138.1).^79^ The feature table was further used for differential abundance analysis using MaAslin2.^44^ In this analysis, the differential abundance testing focused on the genus-level due to resolution of 16S rRNA V4 region; low prevalence genera found in less than 10% of all samples, and genera that were low abundance (average relative abundance below 0.001%) were not included in the testing. Each genus-level was modeled as a function of treatment and sex (categorical variables) with caging as a random effect. Genus levels with a corrected q-value of less than 0.25 are considered significant. 16S rRNA gene amplicon analyses are presented in Supplementary Table S3 and Supplementary TableS 4 and raw sequences were deposited in NCBI SRA, Bioproject ID PRJNA1049485.

### Allobaculum spp *similarity matrix construction*

Using the 16S rRNA V4 sequences from the in house *Allobaculum spp*. we designed the *Allobaculum* specific primers Allo_1_Rev (CCTTTCACTTCAGACTTGCCACG) and Allo_4_For (GGAAACTGTCACGCTCGAGGAC) that we paired, respectively, with 27F and 1492 R bacterial universal primers.^80^ We employed Phusion® High-Fidelity PCR Master Mix with HF Buffer (New England Biolabs) for PCR amplification and Sanger sequencing (Azenta Life Sciences) was performed on each of the PCR amplicons to compile the in-house Allobaculum *spp*. 16S rRNA partial sequence (Supplementary Table S8). Percentage identity matrix for the *Allobaculum spp*. 16S rRNA gene sequences were calculated using ClustalOmega;^48,49^ 16S rRNA gene sequences accession numbers are given in parentheses; *Allobaculum stercoricanis* DSM 13633T (AJ417075), *Allobaculum fili* (MZ153115), and *Allobaculum mucilyticum* (MZ153114).

### Statistical analysis

Results are shown with individual data points and bars indicating mean ± SEM. For comparisons between 2 groups, we use the Mann-Whitney *U* test, and for comparisons between multiple groups a 2-way ANOVA with Benjamini, Kieger and Yekutieli FDR correction for multiple comparisons. *, **, *** and **** represent *p* value or *q* value < 0.05, 0.01, 0.001 or 0.0001, respectively. Statistical analysis was performed using GraphPad Prism (GraphPad) or *R* package for taxonomic analysis.

## Abbreviations

AD: alcohol-dependent
CMT: cecal microbiota transplantation
DSS: dextran sulfate sodium
IBD: inflammatory bowel disease
NIAAA: National institute on Alcohol Abuse and Alcoholism
PMN: polymorphonuclear cell.

## Supplementary Material

Supplemental Material
