# Supplementary material for: Chronic binge drinking-induced susceptibility to colonic inflammation is microbiome-dependent
Source: Gut Microbes. 2024 Aug 20;16(1):2392874. doi: 10.1080/19490976.2024.2392874 (PMC11340762; doi:10.1080/19490976.2024.2392874)
Supplement: Supplemental Material [file KGMI_A_2392874_SM2023.zip › Supplemental Video Links.docx]

**Supplemental Video Link**

<https://drive.google.com/drive/folders/1u7t4V4DRw2Ik7bIRTIC7LK5jX_GnIrUu?usp=drive_link>
