# Supplementary figures and images for: Chronic binge drinking-induced susceptibility to colonic inflammation is microbiome-dependent
Source: Gut Microbes. 2024 Aug 20;16(1):2392874. doi: 10.1080/19490976.2024.2392874 (PMC11340762; doi:10.1080/19490976.2024.2392874)

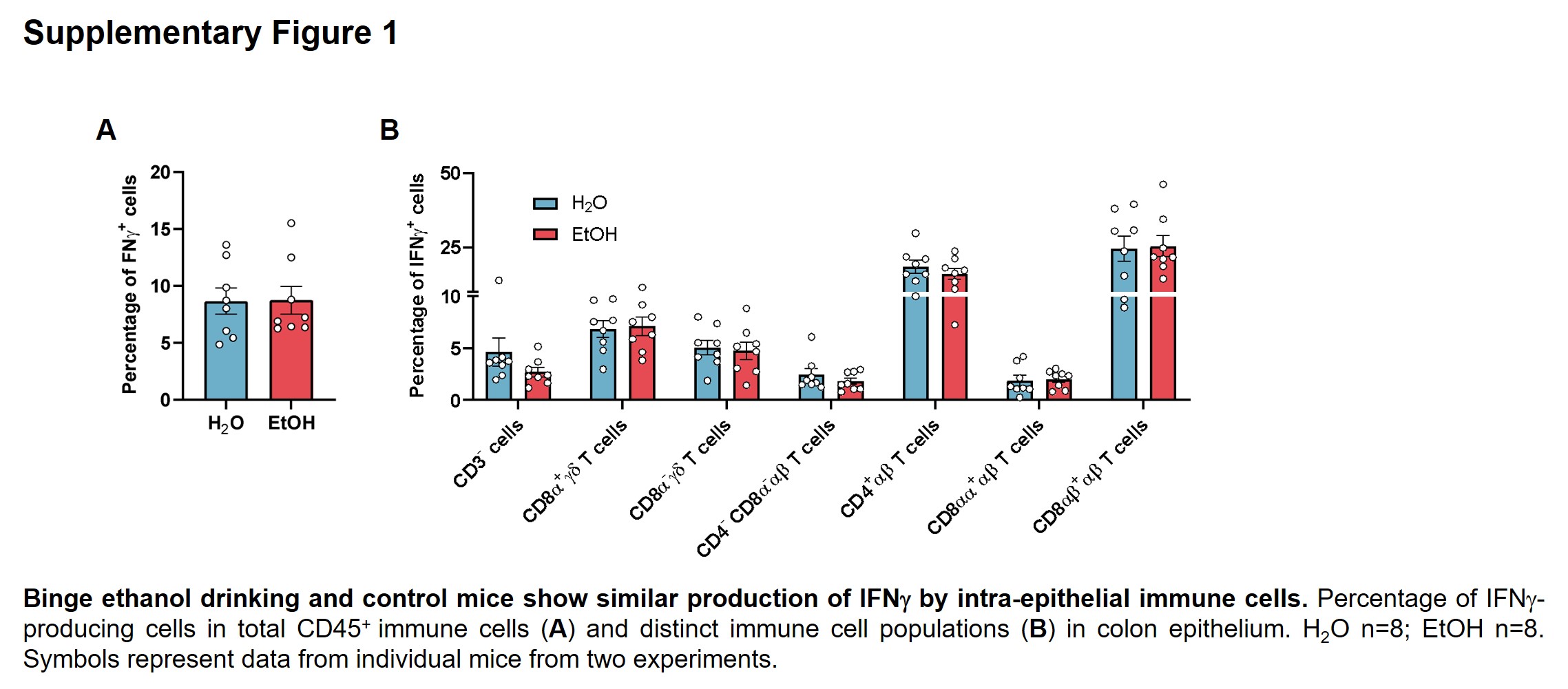

Supplement: Supplemental Material [file KGMI_A_2392874_SM2023.zip › SuppFigure1.jpg]

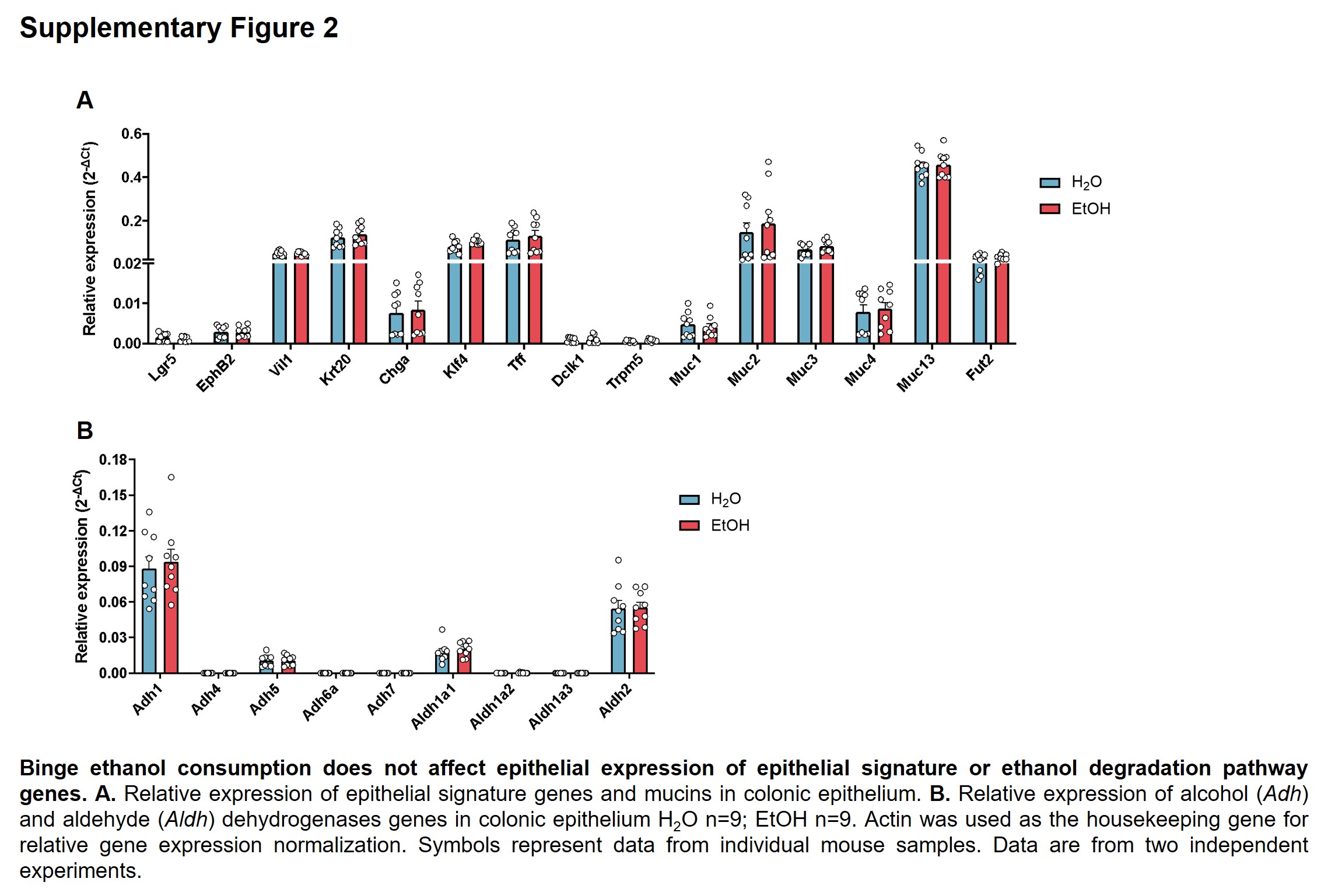

Supplement: Supplemental Material [file KGMI_A_2392874_SM2023.zip › SuppFigure2.jpg]

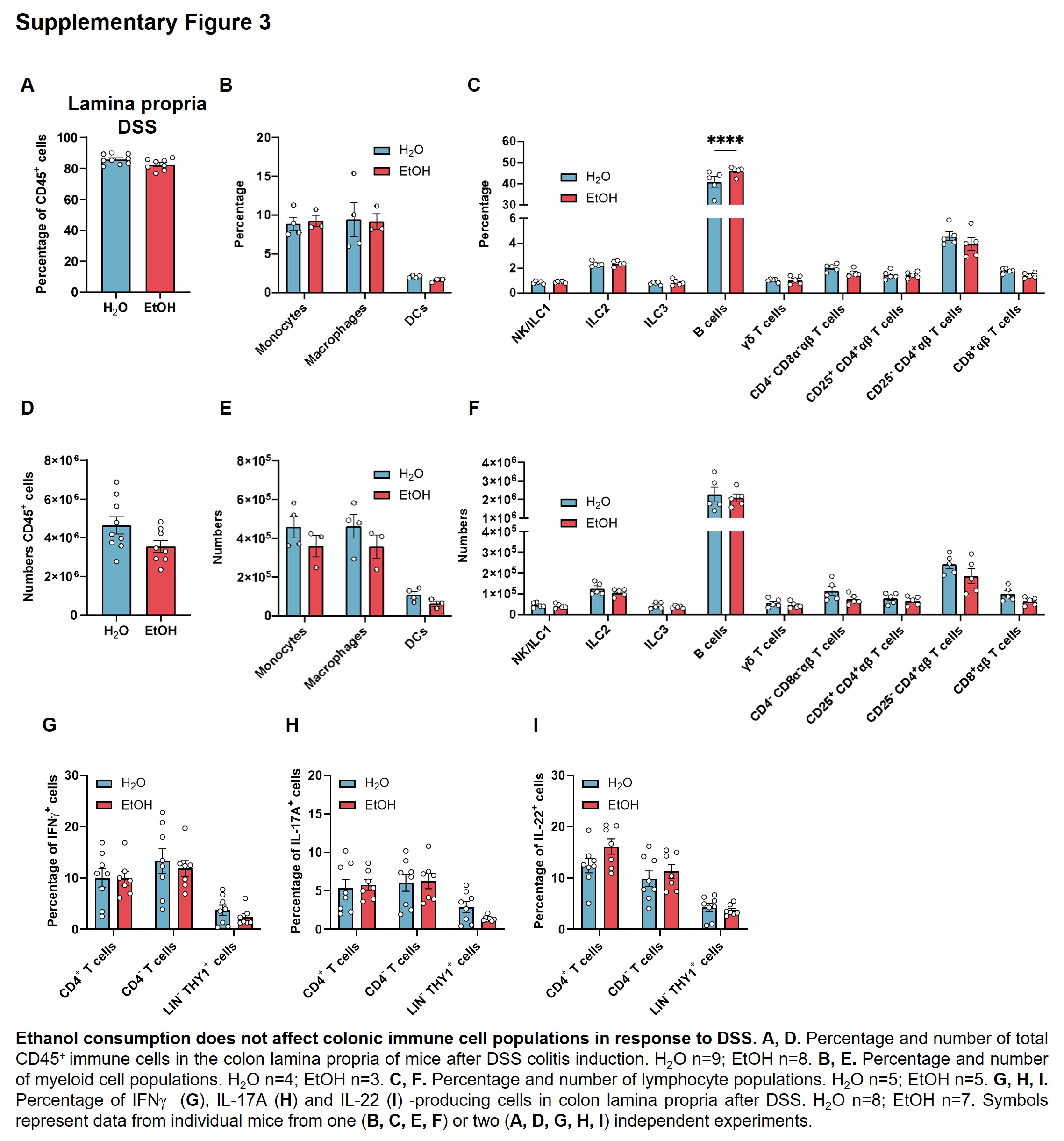

Supplement: Supplemental Material [file KGMI_A_2392874_SM2023.zip › SuppFigure3.jpg]
